# Speckle Tracking Echocardiography Verified the Efficacy of Qianyangyuyin Granules in Alleviating Left Ventricular Remodeling in a Hypertensive Rat Model

**DOI:** 10.1155/2021/5862361

**Published:** 2021-08-24

**Authors:** Anxia He, Lijun Qian, Shihai Yan, Menglin Zhu, Xixuan Zhao, Wenjie Ma, Jie Li, Di Xu

**Affiliations:** ^1^Department of Geriatrics, The First Affiliated Hospital of Nanjing Medical University, Nanjing 210029, China; ^2^Department of Echocardiography, Jiangsu Province Hospital of Chinese Medicine, Affiliated Hospital of Nanjing University of Chinese Medicine, Nanjing 210029, China; ^3^Department of Pharmacology, Jiangsu Province Hospital of Chinese Medicine, Affiliated Hospital of Nanjing University of Chinese Medicine, Nanjing 210029, China; ^4^Department of Cardiology, Jiangsu Province Hospital of Chinese Medicine, Affiliated Hospital of Nanjing University of Chinese Medicine, Nanjing 210029, China

## Abstract

**Background:**

Global longitudinal strain (GLS) can be assessed by speckle tracking echocardiography (STE) to express the degree of cardiac fibrosis. Qianyangyuyin (QYYY) granules can effectively improve GLS in hypertensive patients. Using a hypertensive rat model, we carried out speckle tracking echocardiography to validate the effect of QYYY in diminishing LV remodeling.

**Methods:**

We randomly divided 16 spontaneously hypertensive rats (SHRs) into SHR, SHR + valsartan (SHR + V), SHR + low-dose QYYY (SHR + QL), and SHR + high-dose QYYY (SHR + QH) groups, with four rats in each group. Another group of 4 Wistar-Kyoto (WKY) rats were selected into a normal control (WKY) group. At the 8th week, conventional echocardiographic parameters were measured by GE Vivid E95 ultrasound (12S probe, 10–12 MHz) and GLS by speckle tracking echocardiography with EchoPAC (version 203) software. HE and Masson's trichrome staining were performed to detect the cardiomyocyte width and collagen volume fraction after rat sacrifice. Collagen I, *α*-SMA, S100A4, TGF-*β*, Smad 3, MYH6, and MYH7 were further analyzed by Western blot.

**Results:**

The absolute values of GLS significantly increased in the SHR + QH group compared to the SHR group, while the CVF and CW values significantly decreased. In addition, Collagen I, *α*-SMA, S100A4, TGF-*β*, Smad3, MYH7, and MYH7/MYH6 ratio remarkably reduced in the SHR + QH group. The value of GLS could be repetitively measured and positively correlated with the collagen volume fraction of the myocardium and the cardiomyocyte width of the left ventricular free wall.

**Conclusions:**

GLS is a reliable indicator to evaluate the therapeutic effect on left ventricular remodeling in hypertension. QYYY granules can inhibit the development of cardiac fibrosis in the hypertensive rat model.

## 1. Introduction

Hypertension can cause left ventricular (LV) remodeling [1]. LV remodeling features LV hypertrophy and diffuse interstitial fibrosis [2], both leading to diastolic and systolic dysfunctions [3, 4]. However, how to effectively alleviate LV remodeling and quantify the therapeutic effect noninvasively remains unclear [5].

Speckle tracking echocardiography (STE), a novel echocardiographic imaging technique for myocardial strain analysis [6], can precisely assess LV mechanical systolic function under a hypertensive circumstance [7]. Also, the association between global longitudinal strain (GLS) and cardiac fibrosis has been verified by both clinical and experimental studies [8–10]. By capturing segmental tissue motion on multiple planes and axes serially over the cardiac cycle, strain analysis provides detailed information regarding both regional and global LV functions [11], with much greater sensitivity and specificity than conventional echocardiographic measurements.

Approved by the State Food and Drug Administration of China in 2010, Qianyangyuyin (QYYY) granules have shown the ability of lowering blood pressure, improving renal blood flow, and reducing renal tubulointerstitial diseases [12, 13]. According to pharmacodynamics in spontaneously hypertensive rats [14, 15], QYYY can reduce Ang II in blood and renal tissue and relieve the kidney fibrosis by reducing the expression of TGF-*β*1 to inhibit the activation of myofibroblast. Recently, a double-blind, placebo-controlled, prospective, randomized clinical trial has proved the efficacy and safety of QYYY in more than 107 patients with hypertension [16, 17]. In addition, GLS increased more significantly in QYYY groups.

The aim of this study was to validate the effect of QYYY granules in alleviating left ventricular remodeling in the hypertensive rat model using STE.

## 2. Methods

### 2.1. Reagents and Characterization

Valsartan (batch H20040217) was provided by Beijing Novartis Pharmaceutical Co. Ltd (China). QYYY (batch Z20100007) was obtained from Jiangsu Province Hospital of Chinese Medicine (Nanjing, China). The components of QYYY are listed in Table 1. High-performance liquid chromatogram (HPLC) was used for quality control of QYYY [18]. All the herbs in proportion were soaked with water, followed by heat extraction at 100°C with 10-fold amount of water (weight/volume) twice for 1 hour. The supernatant was filtered and concentrated to a density of 1.2 g/ml. The filtrate was supplemented with stevioside at a concentration of 1% (weight/volume). Having been mixed with dextrin, the granules were granulated in fluidized bed. QYYY granules were dissolved in water and used at an appropriate concentration.

### 2.2. Animals and Ethics

All procedures were approved by the Committee on the Use of Live Animals in Teaching and Research (CULTAR, Approval ID: IACUC-2019DW-07-02) set by the Affiliated Hospital of Nanjing University of Chinese Medicine. All experiments were performed according to the “Principles of Laboratory Animal Care” and the “Guide for the Care and Use of Laboratory Animals” published by the National Institutes of Health (No. 85–23, revised in 1996). A total of 16 spontaneously hypertensive rats (SHR, aged 11 weeks) and other 4 Wistar-Kyoto (WKY, aged 11 weeks) rats weighing 250–300 g were purchased from the Experimental Animal Center of Weitonglihua (SCXK-2012-0001, Beijing, China). The newly arrived animals were kept for at least 10 days in a 12 h/12 h light/dark cycle at 21–23°C and fed with a standard chow diet ad libitum before experiments. Baseline indexes, including body weight, heart rate, and body temperature, were recorded.

### 2.3. Study Design

A total of 16 spontaneously hypertensive rats were randomly assigned to the model control (SHR, *n* = 4), SHR + valsartan (SHR + V, *n* = 4), SHR + low-dose QYYY (SHR + QL, *n* = 4), and SHR + high-dose QYYY (SHR + QH, *n* = 4) groups, and other 4 Wistar-Kyoto rats were selected as a normal control (WKY, *n* = 4) group. QYYY was administered orally for eight consecutive weeks: low dose (5 g/kg/day) in the SHR + QL group and high dose (10 g/kg/day) in the SHR + QH group. Valsartan (positive drug) was administered orally for eight consecutive weeks with a dose of 0.027 g/kg per day in the SHR + V group. WKY and SHR groups received equivalent volume of drinking water in the same way. Additionally, the body weight and blood pressure of rats were recorded before and after the intervention. After that, standard conventional echocardiography parameters were tested and STE was conducted to obtain values of global longitudinal strain (GLS), global circumferential strain (GCS), and global radial strain (GRS). Then, HE and Masson's trichrome staining were performed to detect the cardiomyocyte width (CW) and collagen volume fraction (CVF) after rat sacrifice. Finally, the indicators of cardiac fibrosis were tested by western blot.

### 2.4. Conventional Echocardiography

Routine echocardiography and two-dimensional speckle tracking imaging were performed after eight weeks of intervention, to obtain their left ventricular structural and functional parameters and left ventricular myocardial strain parameters. Rats were anesthetized (intraperitoneal injection of 100 mg/kg ketamine and 20 mg/kg of xylazine) before transthoracic standard echocardiography at a room temperature of 22°C and in a supine left decubitus position by an experienced operator. Echocardiography was performed with a Vivid E95 ultrasound machine (GE Medical Systems, Milwaukee, Wisconsin) equipped with a 12S probe at 10.0–12.0 MHz, as recommended by the American Society of Echocardiography (ASE) [19]. Two-dimensional images of parasternal long-axis and short-axis views and the apical 3-, 4-, and 2-chamber views were recorded, with five consecutive cycles for each view. Left ventricular diastolic diameter (LVDd), left ventricular systolic diameter (LVDs), interventricular septal thickness at diastole (IVSd), and left ventricular postwall thickness at diastole (LVPWd) in parasternal long-axis view were measured, and left ventricular ejection fraction (LVEF) was calculated by the Teich method. The relative wall thickness (RWT) was calculated as the sum of IVSd and LVPWd divided by LVDd. The mitral inflow velocity was obtained from the apical 4-chamber view with a pulsed wave Doppler, and the early peak flow velocity (*E*) was recorded. Tissue Doppler imaging was performed to measure mitral annulus excursion. Early diastolic peak velocity (*e*) of the septal corner of the mitral annulus was recorded. The *E*/*e* ratio was calculated.

### 2.5. Speckle Tracking Echocardiography (STE)

**Figure 1:**
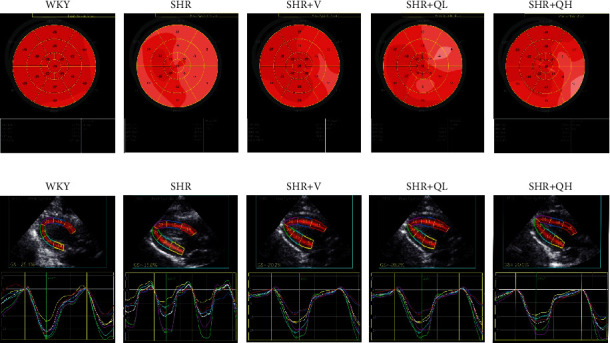
Representative pictures of (a) the 17-segment bull's eye diagram and (b) longitudinal strain diagram of the apical 3-chamber view among 5 groups following speckle tracking echocardiography. Bull's eye diagram (a) visually showed the global and 17-segmental longitudinal strains of LV. Red indicates the normal segmental strain value (absolute value), while the lighter color means the lower strain value. The lower the absolute value, the worse the mechanical systolic function of the cardiac segment. The bull's eye diagram showed uniformly red in the WKY group, but many light red regions in the SHR group. The GLS value below the diagram was the average value of the 17-segmental strain in the bull's eye diagram. Longitudinal strain diagram (b) showed the 6-segmental strain of a certain chamber (apical 2-, 3-, and 4-chamber views) and the strain curve of each segment with time. The strain curve of each segments was close to the same in WKY rats.

Using EchoPAC software (version 203, GE Healthcare, Horten, Norway), STE was performed offline for GLS, GCS, and GRS analysis. For the longitudinal speckle tracking analysis, three endocardial markers were placed in an end-diastolic frame at the apical 3-chamber view. The software automatically tracked and contoured the area of interest between endocardial and epicardial borders. Subsequently, grayscale images were analyzed, followed by frame-to-frame movement of stable patterns of natural acoustic markers (or speckles). The 6-segmental strain of the chamber and the strain curve of each segment with time were obtained (Figure 1(b)), after confirming the time of aortic valve closure. The apical 2- and 4-chamber views were analyzed. Then, the 17-segment bull's eye diagram of LV GLS was drawn with speckle tracking imaging (Figure 1(a)), and GLS (negative value) was calculated as the average value of the 17-segmental strain in the bull's eye diagram. The same procedure was used for the short-axis view at the level of papillary muscle of the left ventricle to obtain GCS (negative value) and GRS. Finally, the GLS, GCS, and GRS were analyzed by two independent investigators.

### 2.6. Pathological Analysis

Masson's trichrome staining was performed to observe cardiac fibrosis. After rat sacrifice, the CVF of endocardium, midmyocardium, and epicardium, as well as perivascular collagen area/vascular lumen area ratio (PVCA/LA), were analyzed and calculated by Image J64 software. Under a 400x light microscope, five field images of the endocardium, midmyocardium, and epicardium from each heart were randomly taken to measure the fibrotic area, respectively. CVF was calculated as the percentage of collagen fiber area in myocardial tissue. The cross sections of 5 arterioles selected from each heart were photographed. Then PVCA/LA value was obtained using the same method. Hematoxylin and eosin (HE) staining of myocardial tissues was done to observe cardiomyocytes, and CW of the septal and free wall was measured by Image J64 software. Twenty cardiomyocytes of each wall were measured under a 400x light microscope.

### 2.7. Western Blotting

The whole tissue lysate (50 *μ*g) was separated by SDS-PAGE (8% and 10% half-half layered) and transferred to PVDF membranes (0.25 *μ*m). Having been blocked in 5% BSA at room temperature for 1 hour, the membranes were incubated with proper primary antibodies at 4°C overnight. Then, secondary antibodies were used to incubate the membranes at 4°C for 2 hours. With a potent chemiluminescence kit and super signal west femto trail kit, the expression of target proteins (Collagen I, *α*-SMA, S100A4, TGF-*β*, Smad3, MYH6, and MYH7) were finally detected.

### 2.8. Quantitative RT-PCR (qRT-PCR)

Total RNA was extracted from samples by TRIzol and then reverse-transcribed using a PrimeScript™ RT reagent kit for cDNA synthesis. We used a SYBR Premix Ex Taq™ II kit for miRNA detection. Gene specific primers were used to amplify S100A4 (forward: AGCACTTCCTCTCTCTTGGTC; reverse: GTCTGTCCTTCTCCCCAGGA) and GAPDH (forward: TGCCACTCAGAAGACTGTGG; reverse: TTCAGCTCTGGGATGACCTT). In PCR, preincubation at 95°C for 30 seconds was first performed, followed by 40 cycles of denaturation (95°C for 5 seconds) and annealing (60°C for 30 seconds) and a final dissociation. A StepOnePlus Real-Time PCR System was used to perform qRT-PCR.

### 2.9. Statistical Analysis

All experiments were repeated to obtain with repetitions qualitatively similar data. All data were analyzed by SPSS 17.0 software (SPSS Inc, Chicago, Illinois, 2008). Values were expressed as mean ± standard deviation. Intraobserver and interobserver variabilities (Bland–Altman plots) were analyzed by GraphPad Prism Version 6. Intraclass correlation coefficients (ICCs) and coefficient of variation (CV) were calculated by SPSS Version 24. The between-group difference was analyzed with unpaired Student's *t*-test or one-way ANOVA test. All statistical tests were two-sided, and *p* < 0.05 was considered statistically significant.

## 3. Results

### 3.1. Conventional Echocardiography Parameters Improved in QH-Treated SHRs

Conventional echocardiography parameters were obtained to evaluate the structure and function of LV (Table 2). The IVSd and LVPW in SHR + QH and SHR + QL groups and the IVSd in the SHR + V group were significantly lower than those in the SHR group. In addition, the LVM and RWT in the SHR + QH group were significantly lower than those in the SHR group. LVEF significantly decreased in the SHR group, compared with the WKY group, while there was no statistical difference in the *E*, *e*, and *E*/*e*. The LVEF and *e* in the SHR + QH group were significantly higher, while its *E*/*e* was significantly lower than that in SHR and SHR + QL groups. The LV systolic function and hypertrophy in hypertensive rats improved in the QH-treated group.

### 3.2. GLS Increased in QH-Treated SHRs

**Figure 2:**
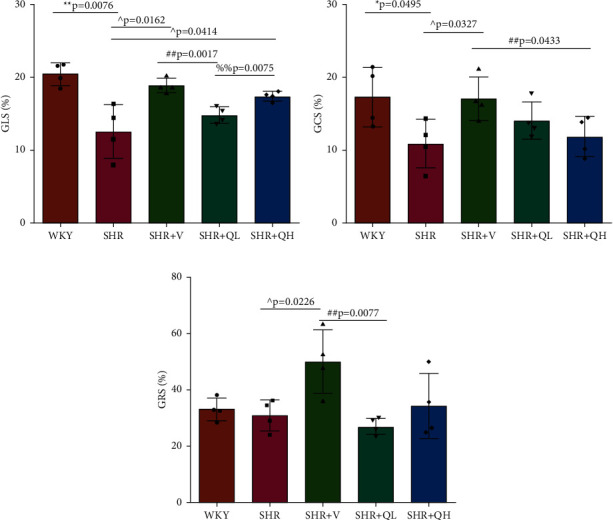
The absolute value of (a) GLS significantly increased in high-dose QYYY-treated SHRs, comparing with the SHR group. The absolute values of (b) GCS in SHR + V and SHR + QH groups were higher than those in the SHR group, but the significant difference only showed in the SHR + V group, as well as (c) GRS. Values are means ± SD. GLS, global longitudinal strain; GCS, circumferential strain; GRS, global radial strain; WKY, Wistar-Kyoto; SHR, spontaneously hypertensive rats; SHR+(V) SHR + valsartan; SHR + QL, SHR + QYYY low dose; SHR + QH, SHR + QYYY high dose. ^*∗*^*P* < 0.05 and ^*∗∗*^*P* < 0.01, compared to the WKY group;  ^∧^*P* < 0.05 and  ^∧∧^*P* < 0.01, compared to the SHR group; ^##^*P* < 0.01, compared to the SHR + V group; ^%%^*P* < 0.01, compared to the SHR + QL group.

GLS was measured to evaluate LV mechanical function in hypertensive rats treated with and without QYYY (Figure 2(a)). Among 340 myocardial segments, 18 segments were poorly tracked, mainly distributed in LV mid-wall and basal segments. The absolute values of LV GLS in SHR + V and SHR + QH groups were significantly higher than those in the SHR group and significantly lower than those in the WKY group. However, there was no significant difference in GLS between SHR + V and SHR + QH groups. The changes in GLS values among groups were consistent with those in LVEF. The results showed a good efficiency of GLS analysis to assess LV mechanical systolic function after QYYY treatment.

### 3.3. GCS and GRS Changed in QH-Treated SHRs

GCS and GRS were also measured to detect LV systolic function. As shown in Figure 2(b), the absolute values of GCS in the SHR + V group is significantly higher than those in the SHR group, but there was no significant improvement in QYYY groups. GRS values in SHR + V and SHR + QH groups were higher than those in the SHR group, but the significant difference only showed in the SHR + V group (Figure 2(c)). The results showed a poor efficiency of GCS and GRS to assess LV systolic function after both high- and low-dose QYYY treatment.

### 3.4. GLS Measurements Had a Good Repeatability

**Figure 3:**
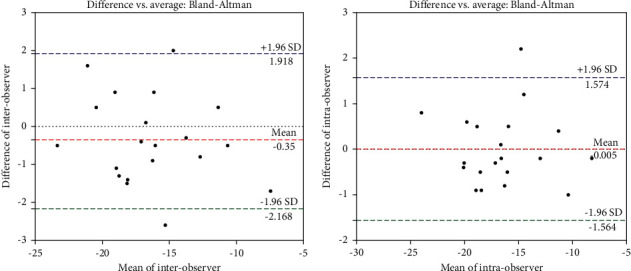
Interobserver and intraobserver variability. (a) Bland–Altman plots showing a high agreement between 2 repeated measurements of GLS performed by both observers. (b) Bland–Altman plots showing intraobserver agreement on GLS measurements.

As shown by the ICCs (0.98 and 0.97) in Table 3, a strong correlation and a high agreement were observed between intraobserver and interobserver GLS values. The intraobserver and interobserver CV values were higher in GRS (17.65 ± 6.95% and 11.79 ± 4.77%) and GCS (10.85 ± 1.53% and 7.77 ± 1.12%) than those in GLS (3.35 ± 0.55% and 4.39 ± 0.71%), with poor repeatability. In apical long-axis views, the direction of myocardial movement was consistent with that indicated by the ultrasound. When the apical long-axis views were used to measure GLS, it may be more accurate to identify the endocardial boundaries and track the myocardial movement. However, when the short-axis view of left ventricular papillary muscle level was used to measure GCS and GRS, the hypertrophic papillary muscle and trabecula made it difficult to trace the endocardium accurately, which may affect the tracking and analysis. In addition, Bland–Altman plots were employed to determine GLS agreements. There was a high agreement between 2 repeated measurements performed by both observers (Figure 3(a)), and there was a very strong correlation for all intraobserver measurements and the correlation was statistically significant (Figure 3(b)).

### 3.5. QYYY Granules Improved Blood Pressure in SHRs

**Figure 4:**
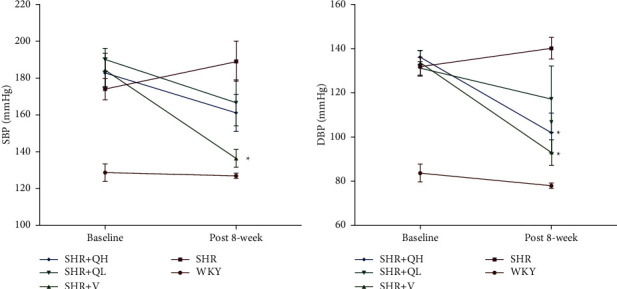
QYYY granules improved blood pressure in SHRs. Compared to the SHR group, (a, b) the systolic and diastolic blood pressure significantly reduced in the SHR + V group. (b) The diastolic blood pressure was significantly lower in the SHR + QH group than in the SHR group (a, b) while there was no statistical difference between SHR and SHR + QL groups. ^*∗*^*P* < 0.05, compared to the baseline.

Blood pressure before and after QYYY treatment was detected to evaluate the hypotensive effect at baseline and 8 weeks after treatment (Figure 4). The systolic and diastolic blood pressures were significantly higher in all SHR groups than in the WKY group at baseline, while there were no statistical differences among SHR groups. In addition, the systolic and diastolic blood pressures significantly reduced in the SHR + V group, compared to the SHR group (Figures 4(a) and 4(b)). The diastolic blood pressure was significantly lower in the SHR + QH group than in the SHR group (Figure 4(b)), while there was no statistical difference between SHR and SHR + QL groups. QYYY showed a less effect on lowering blood pressure in SHR, compared to valsartan.

### 3.6. QYYY Granules Alleviated LV Remodeling in SHRs

**Figure 5:**
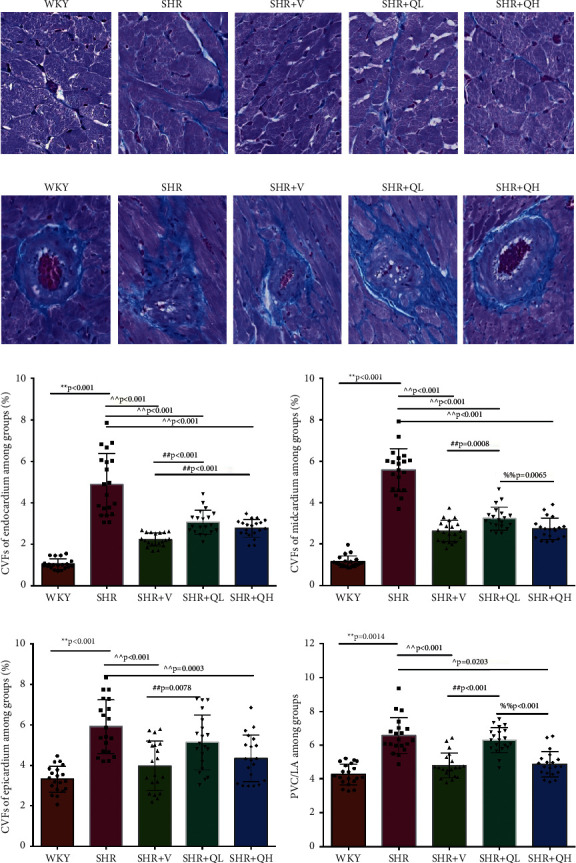
QYYY granules alleviated cardiac fibrosis via decreasing (a) the myocardial interstitial collagen fibers and (b) the collagen fibers around small blood vessels with Masson's trichrome staining analysis.(c), (d) and (e) Comparisons of collagen volume fraction (CVF) and (f) perivascular collagen area/vascular lumen area ratio (PVCA/LA) among the 5 groups. ^*∗∗*^*P* < 0.01, compared to the WKY group;  ^∧^*P* < 0.05 and  ^∧∧^*P* < 0.01, compared to the SHR group; ^##^*P* < 0.01, compared to the SHR + V group; ^%%^*P* < 0.01, compared to the SHR + QL group. *n* = 4 per group.

To detect the efficacy of QYYY granules in attenuating hypertensive cardiac remodeling, Masson's trichrome staining was performed after rat sacrifice (Figure 5). The CVF and PVCA/LA were measured. Under a 400-fold light microscope, the myocardial interstitial collagen fibers and collagen fibers around small blood vessels in SHR + QH and SHR + V groups significantly decreased (Figures 5(a) and 5(b)), compared to those in the SHR group. The CVFs of endocardium, midmyocardium, and epicardium in the SHR + QH group were significantly higher than those in the WKY group but lower than those in the SHR group (Figures 5(c)–5(e)). The PVCA/LA in the SHR + QH group was significantly higher than that in the WKY group but lower than that in the SHR group (Figure 5(f)). However, there was no significant difference in CVFs and PVCA/LAs between SHR + V and SHR + QH groups. The results revealed the significant effect of QYYY granules in alleviating LV remodeling caused by hypertension.

**Figure 6:**
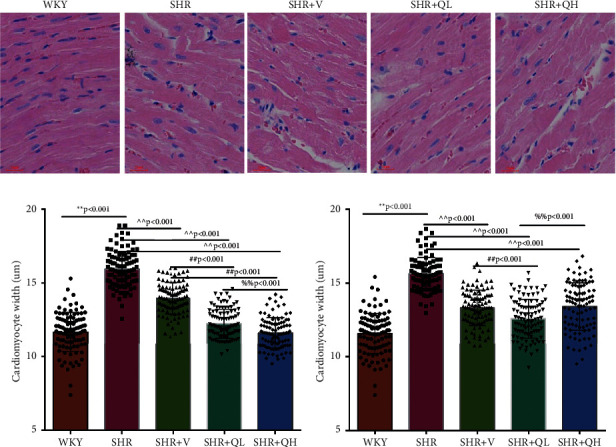
QYYY granules alleviated cardiac hypertrophy according to (a) HE staining and (b), (c) comparison of the left ventricular septum and free wall cardiomyocyte width (CW). ^*∗∗*^*P* < 0.01, compared to the WKY group;  ^∧∧^*P* < 0.01, compared to the SHR group; ^##^*P* < 0.01, compared to the SHR + V group; ^%%^*P* < 0.01, compared to the SHR + QL group. *n* = 4 per group.

HE staining and CW comparison were also used to verify this effect (Figure 6). HE staining showed the cardiomyocytes in regular arrangement and the nuclei of equal size in the WKY group and hypertrophy, structural disorder, and irregular nuclei in the SHR group (Figure 6(a)). The hypertrophy diminished and cardiomyocytes became more neatly arranged in SHR + V and SHR + QH groups. Then, the CWs of LV septum and free wall were significantly lower in SHR + QH and SHR + V groups than in the SHR group, respectively (Figures 6(b) and 6(c)).

### 3.7. QYYY Granules Reduced the Expression of Cardiac Fibrosis-Related Molecules

**Figure 7:**
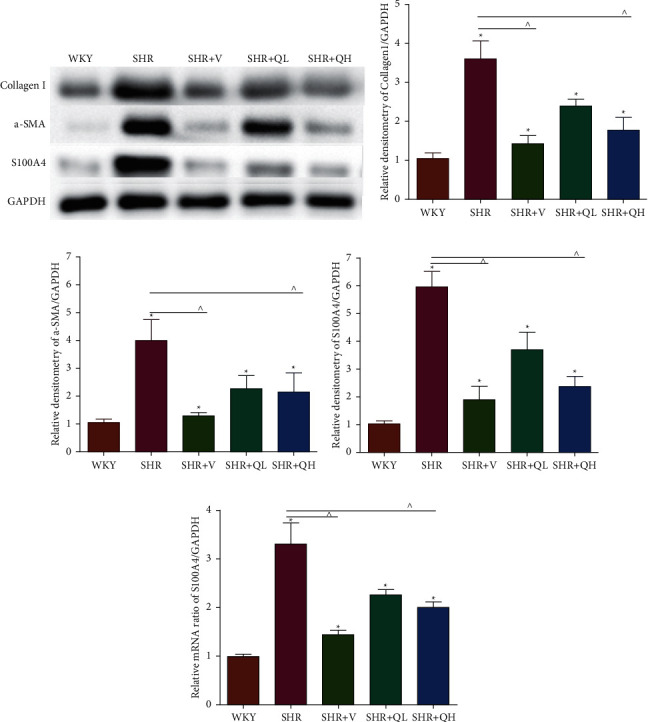
QYYY granules reduced the indicators of cardiac fibrosis. (a, b) The expression of Collagen I and (a, c) *α*-SMA proteins significantly reduced in the SHR + QH group when compared with the SHR group by western blot. (a, d) S100A4 protein expression also significantly reduced in the SHR + QH group. According to the results of (e) qRT-PCR, the mRNA expression of S100A4 decreased dramatically in SHR + V and SHR + QH groups. ^*∗*^*P* < 0.05, compared to the WKY group;  ^∧^*P* < 0.05, compared to the SHR group. *n* = 4 per group.

The levels of Collagen I (Figures 7(a) and 7(b)) and *α*-SMA (Figures 7(a) and 7(c)) expression obviously dropped in the SHR + QH and SHR + V groups when compared with the SHR group tested by western blot. According to western blot, the level of S100A4 (also named fibroblast-specific protein 1) (Figures 7(a) and 7(d)) dramatically decreased in the SHR + QH and SHR + V groups when compared with the SHR group. According to the results of qRT-PCR (Figure 7(e)), the mRNA expression of S100A4 decreased dramatically in SHR + V and SHR + QH groups, which proved the successful downregulation of S100A4 in the hypertensive rat model. The results highlighted that QYYY granules inhibited myofibroblast differentiation and proliferation in the hypertensive rat model.

### 3.8. QYYY Granules Downregulated Cardiac Fibrosis and Hypertrophy

**Figure 8:**
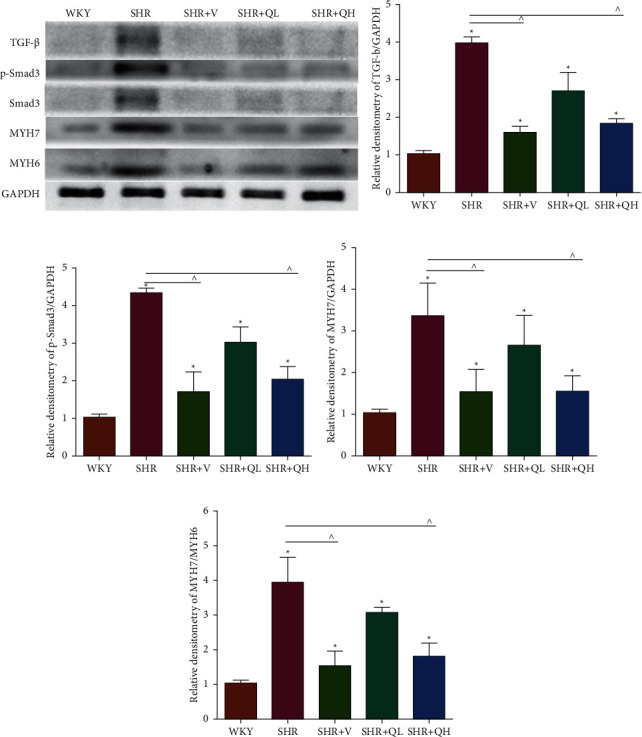
QYYY granules downregulated cardiac fibrosis and hypertrophy. Comparing with the SHR group, the expression of (a, b) TGF-*β* and (a, c) p-Smad 3 proteins, which were the key genes induced in the cardiac fibrosis, significantly reduced in the SHR + QH group by western blot. QYYY granules reduced the level of (a, d) MYH7 and (a, e) the rate of MYH7/MYH6 involved in cardiac hypertrophy in the SHR + QH group when compared with the SHR group by western blot. ^*∗*^*P* < 0.05, compared to the WKY group;  ^∧^*P* < 0.05, compared to the SHR group. *n* = 4 per group.

Comparing with the SHR group, the expression of TGF-*β* (Figures 8(a) and 8(b)) and p-Smad 3 (Figures 8(a) and 8(c)) proteins significantly reduced in the SHR + QH and SHR + V groups tested by western blot. QYYY granules also lowered the level of MYH7 (Figures 8(a) and 8(d)) and the ratio of MYH7/MYH6 (Figures 8(a) and 8(e)) in the SHR + QH and SHR + V groups when compared with the SHR group. It demonstrated that QYYY granules can downregulate the key genes involved in cardiac fibrosis and cardiac hypertrophy.

### 3.9. LV Remodeling Severity Was Positively Correlated with GLS Values

The values of GLS and GCS were significantly positively correlated with the CVFs of LV endocardium, midmyocardium, and epicardium, respectively (Table 4). The GLS value was also positively correlated with the PVCA/LA. The GRS value was positively correlated with LVEF. In addition, the values of GLS and GCS were significantly positively correlated with the CWs of LV free wall, but not with the CWs of LV septum and conventional echocardiography parameters.

## 4. Discussion

In this study, the efficiency of 17-segmental GLS in evaluating LV remodeling in a hypertensive rat model was first evaluated. First, STE showed the favorable efficiency of GLS in evaluating LV mechanical systolic function in SHRs treated with high-dose QYYY granules. The conventional echocardiography was used to verify the accuracy of GLS in evaluating LV systolic function in the SHR + QH group. Repeatability of strain measurements and correlations between cardiac remodeling and GLS were detected. In addition, the effect of QYYY granules in alleviating LV remodeling in a hypertensive rat model was further evaluated. CVF and CW comparison results confirmed the efficacy of QYYY granules in alleviating LV remodeling. The myocardial fibrosis-related factors detected by western blot further confirmed that QYYY granules can downregulate the key genes involved in hypertensive myocardial remodeling. As a result, GLS can be used as a new index for evaluating the efficacy of drug intervention in LV remodeling and QYYY granules were able to improve LV systolic function and remodeling in SHRs.

Late-gadolinium-enhanced cardiac magnetic resonance (CMR) sequence and T1-map technology have been tried for noninvasive quantitative detection of cardiac remodeling [20, 21]. However, CMR is expensive, time-consuming, and patient-cooperation-needed, and it is not convenient for clinical follow-up evaluation. The two-dimensional STE is derived from the conventional echocardiography. By acquiring two-dimensional dynamic images, GLS, GCS, and GRS can be measured quickly by STE. Clinical myocardial biopsy has found that GLS is significantly related to cardiac remodeling [8], and Ishizu's study has confirmed that strain value is correlated with the degree of LV remodeling in hypertension with LV GLS, GCS, and GRS values tracked and analyzed in SHRs [10]. However, in the study, the longitudinal strain measured by the apical 3-chamber view was used as the global longitudinal strain of the left ventricle. 17-segmental GLS has been widely used to evaluate hypertensive injury in clinical research [7, 11, 22], but its indicative value for cardiac remodeling and drug intervention has not been reported.

The 17-segment analysis method recommended by ASE was used to calculate the GLS of LV and to obtain the bull's eye diagram on the planes of apical 3-, 4-, and 2-chamber views. In addition, the GCS and GRS on the LV short-axis view were measured. Among 340 myocardial segments analyzed for GLS, 18 segments were poorly tracked and distributed in the LV mid-wall and basal segments, the proportion of which is similar to that in the study of Barbier et al. [23]. Pearson correlation analysis found that GLS was positively correlated with the CVF of each myocardium layer, which is consistent with the results of Leader et al. [24]. In addition, GLS and CVF showed a consistent trend among all groups. GCS was significantly positively correlated with the CVF of each myocardium, but it was significantly improved only in the SHR + V group with normal blood pressure after treated, which indicated that GCS may be associated with cardiac afterload. The repeatability test revealed a good repeatability of GLS in both intraobservers and interobservers. These results demonstrate that GLS can be used to describe the degree of LV remodeling in a given time period and to evaluate the efficacy of clinical drug intervention.

The SHR group showed higher LVM, IVSd, and RWT and lower LVEF than the WKY group, indicating that a SHR model had been successfully established. The antihypertensive effect of QH administration was lower than that of valsartan, but its effect of improving myocardial remodeling had no significant difference from that of valsartan, which is consistent with previous clinical reports. These results demonstrate that QYYY granules have an ability of improving myocardial remodeling independent of antihypertension. QYYY granules can substitute for valsartan to lower blood pressure and alleviate LV remodeling.

Our previous clinical trials have demonstrated the efficacy of QYYY granules in safeguarding cardiac function. QYYY granules can also counter renal fibrosis by inhibiting the production of TGF-*β* [15], regulating the AngII-AT1R-CTGF pathway [13], and repressing the NF-*κ*B inflammation pathway and the expression of NOX4 [12]. Relevant studies hold that these pathways are also implicated in hypertensive cardiac fibrosis [25, 26]. In the process of myocardial remodeling in hypertension, TGF-*β* is activated by factors such as angiotensin II and endothelin-1 and then regulates downstream pathways like Smad2/3, to promote fibroblast proliferation and phenotypic transformation into myofibroblast and then to increase gene expression of Collagen I and III [25, 27]. *α*-Smooth muscle actin (*α*-SMA) is widely used to mark the activation status of fibroblasts that differentiate into myofibroblasts [28]. Studies have found that S100A4 (fibroblast-specific protein 1) is associated with myocardial fibrosis after myocardial infarction [29] and is regulated by Smad3 in myocardial interstitial fibroblasts [30], promoting myocardial fibrosis in the mouse model induced by pressure overload [31]. In this study, the level of S100A4, Collagen I, and *α*-SMA dramatically decreased after QH administration, which highlighted that QYYY granules inhibited myofibroblast differentiation in the hypertensive rat model. The expression of TGF-*β* and p-Smad3 reduced after QYYY treatment, as well as MYH7 and MYH7/MYH6, which demonstrated QYYY granules can downregulate the key genes involved in cardiac fibrosis and cardiac hypertrophy. Whether QYYY granules interfere with cardiac fibrosis in LV remolding through the TGF-*β*/Smad3 pathway to regulate the expression of S100A4 remains to be verified.

To sum up, this study verified GLS can be used to evaluate hypertensive LV remodeling and the effect of intervention. QYYY granules can alleviate left ventricular remodeling without the need of lowering blood pressure. Further trials are needed to prove the clinical usefulness of STE in evaluating LV remodeling.

### 4.1. Limitations

Limitations are as follows: (1) Rats have higher heart rates and different hemodynamics in comparison with humans. Models constructed with pigs or canines are needed to fortify our finding. (2) Because of the small size of our experiment and the loss of baseline charterers, the potential of STE in strain detection should be more exploited. (3) The underlying mechanism through which QYYY granules hinder LV remodeling through inhibiting cardiomyocyte apoptosis remains to be determined.

## 5. Conclusion

High-dose QYYY granules can alleviate LV remodeling in SHRs. GLS can assess the efficacy and safety following QYYY administration.

## Figures and Tables

**Table 1:** The components of QYYY.

Components	Chinese name	Amount used (g)
Herba Bidentis Bipinnatae	Gui Zhen Cao	110
Polygoni Multiflori Radix	He Shou Wu	180
Corni Fructus	Shan Zhu Yu	108
Cyathulae Radix	Chuan Niu Xi	170
Scrophulariae Radix	Xuan Shen	180
Alismatis Rhizoma	Ze Xie	180

**Table 2:** Comparison of LV structural and functional parameters among 5 groups.

Group	LVM (g)	LVD (mm)	IVSd (mm)	LVPW (mm)	RWT	LVEF (%)	E (cm/s)	e (cm/s)	E/*e*
WKY (*n* = 4)	1.18 ± 0.06	6.68 ± 0.24	1.68 ± 0.19	1.59 ± 0.08	0.50 ± 0.05	79.75 ± 2.99	107.50 ± 10.75	4.00 ± 0.82	27.36 ± 3.32
SHR (*n* = 4)	1.33 ± 0.12^*∗∗*^	6.18 ± 0.34	2.32 ± 0.08^*∗∗*^	1.91 ± 0.12^*∗∗*^	0.68 ± 0.03^*∗∗*^	73.75 ± 3.30^*∗∗*^	97.75 ± 19.62	3.25 ± 0.50	30.73 ± 8.42
SHR + V (*n* = 4)	1.24 ± 0.02	6.14 ± 0.47^*∗∗*^	2.07 ± 0.14^*∗∗*∧^	1.96 ± 0.05^*∗∗*^	0.66 ± 0.07^*∗∗*^	85.50 ± 1.29^*∗∗*∧∧^	81.00 ± 8.83^*∗*^	4.12 ± 0.25	19.69 ± 2.48^∧^
SHR + QL (*n* = 4)	1.24 ± 0.04	6.28 ± 0.47	1.99 ± 0.07^*∗∗*∧∧^	1.73 ± 0.17^∧∧#^	0.62 ± 0.07^*∗*^	75.75 ± 1.50^*∗*##^	107.50 ± 19.60^#^	3.75 ± 0.96	29.29 ± 4.81^#^
SHR + QH (*n* = 4)	1.19 ± 0.06^∧#%^	5.71 ± 0.35^∧∧^	1.85 ± 0.05^∧∧#%^	1.65 ± 0.07^∧∧#^	0.61 ± 0.05^*∗*∧^	83.25 ± 3.30^∧∧%%^	90.50 ± 19.69	5.50 ± 1.73^*∗*∧∧%^	17.59 ± 6.01^*∗*∧%^

Values area means ± SD. WKY, Wistar-Kyoto; SHR, spontaneously hypertensive rats; SHR + V, SHR + valsartan; SHR + QL, SHR + QYYY low dose; SHR + QH, SHR + QYYY high dose; LVM, left ventricular mass; LVD, left ventricular internal dimension at end diastole; IVSd, interventricular septal thickness at diastole; LVPW, left ventricular postwall thickness at diastole; RWT, relative wall thickness; LVEF, left ventricular ejection fraction. ^*∗*^*P* < 0.05 and ^*∗∗*^*P* < 0.01, compared to the WKY group; ^∧^*P* < 0.05 and ^∧∧^*P* < 0.01, compared to the SHR group; ^#^*P* < 0.05, compared to the SHR + V group; ^%^*P* < 0.05 and ^%%^*P* < 0.05, compared to the SHR + QL group.

**Table 3:** Intraobserver and interobserver differences among GLS, GCS, and GRS measurements.

Group	Intraobserver		Interobserver	
	ICC (*n* = 20)	CV (*n* = 20)	ICC (*n* = 20)	CV (*n* = 20)
GLS (%)	0.98	3.35 ± 0.55%	0.97	4.39 ± 0.71%
GCS (%)	0.85	10.85 ± 1.53%	0.92	7.77 ± 1.12%
GRS (%)	0.65	17.65 ± 6.95%	0.86	11.79 ± 4.77%

Values area means ± SD. GLS, global longitudinal strain; GCS, circumferential strain; GRS, global radial strain. ICC, intraclass correlation coefficients; CV, coefficient of variation.

**Table 4:** Correlation between LV remodeling and LV strain.

Parameters	GLS	GCS	GRS
LV endocardium CVF	0.958^*∗*^	0.923^*∗*^	−0.32
LV midmyocardium CVF	0.936^*∗*^	0.884^*∗*^	−0.262
LV epicardium CVF	0.973^*∗∗*^	0.959^*∗*^	0.6
PVCA/LA	0.873^*∗*^	0.76	−0.044
LV septum CW	0.787	0.681	0.071
LV free wall CW	0.950^*∗*^	0.944^*∗*^	−0.619
LVD	−0.148	−0.105	−0.45
IVSd	0.802	0.685	0.072
RWT	0.624	0.494	0.326
LVEF	−0.763	−0.798	0.898^*∗*^
*E*	0.162	0.269	−0.853
*e*	−0.456	−0.368	0.196
*E*/*e*	0.351	0.301	−0.226

GLS, global longitudinal strain; GCS, global circumferential strain; GRS, global radial strain. CVF, collagen volume fraction; CW, cell width; PVCA/LA, perivascular collagen area/vascular lumen area ratio; LVD, left ventricular internal dimension at end diastole; IVSd, interventricular septal thickness at diastole; RWT, relative wall thickness; LVEF, left ventricular ejection fraction. ^*∗*^*P* < 0.05 and ^*∗∗*^*P* < 0.01, compared between LV remodeling and LV strain.

## Data Availability

All procedures were approved by the Committee on the Use of Live Animals in Teaching and Research (CULTAR, Approval ID: IACUC-2019DW-07-02) set by the Affiliated Hospital of Nanjing University of Chinese Medicine.
